# Prevalence of Metabolic Syndrome and its influence on microvascular complications in the Indian population with Type 2 Diabetes Mellitus. *Sankara Nethralaya Diabetic Retinopathy Epidemiology And Molecular Genetic Study (SN-DREAMS, report 14)*

**DOI:** 10.1186/1758-5996-2-67

**Published:** 2010-11-11

**Authors:** Rajiv Raman, Aditi Gupta, Swakshyar S Pal, Suganeswari Ganesan, Kadri Venkatesh, Vaitheeswaran Kulothungan, Tarun Sharma

**Affiliations:** 1 Shri Bhagwan Mahavir Vitreoretinal Services, 18, College Road, Sankara Nethralaya, Chennai-600 006, Tamil Nadu, India; 2 Department of Preventive Ophthalmology (Epidemiology and Biostatistics), 18, College Road, Sankara Nethralaya, Chennai-600 006, Tamil Nadu, India

## Abstract

**Background:**

The Metabolic syndrome (MS) consists of central obesity, glucose intolerance, hyperinsulinemia, low high density lipoproteins, high triglycerides and hypertension. Different studies have observed that MS causes microvascular complications in patients with type 2 diabetes. The aim of the study was to find out the prevalence of MS in the Indian population with type 2 diabetes mellitus in relation to gender, duration of diabetes, and to evaluate the influence of MS and its individual components on microvascular complications such as diabetic retinopathy, diabetic nephropathy and diabetic neuropathy.

**Methods:**

A population-based cross sectional survey was conducted with 1414 patients having type 2 diabetes mellitus. The International Diabetes Federation (IDF) criteria were used to identify the metabolic syndrome. Diabetic retinopathy was graded using the stereoscopic digital fundus photography. Neuropathy was assessed by measuring the vibration perception threshold through a sensitometer. Nephropathy was diagnosed by the presence of microalbuminuria in the first morning urine sample.

**Results:**

The age and gender adjusted prevalence of MS, using the IDF criteria, in the South Indian population was 73.3%. The prevalence was higher in women (83.3%), compared to men (65.3%). In subjects with diabetes mellitus, without and with MS, the prevalence of retinopathy was 21.3% and 16.9% (p = 0.057); prevalence of nephropathy was 20.5% and 18.0% (p = 0.296), and prevalence of neuropathy was17.2% and 19.4% (p = 0.353) respectively. Overall and in women, the clustering of MS components led to an increase in the prevalence of diabetic nephropathy. The prevalence of retinopathy and neuropathy in MS subjects, who had diabetes for < 10 years, was more in both men and women; it was more in women but not in men when the duration of diabetes varied from 11-20 years.

**Conclusions:**

The association of MS with microangiopathies decreased with an increase in the duration of diabetes. MS behaved differently in men and women. It may need to be managed differently in the two groups.

## Background

The Metabolic Syndrome (MS) is a constellation of central obesity, glucose intolerance, hyperinsulinemia, low high density lipoproteins (HDL), high triglycerides and hypertension [1]. It is associated with a high risk of coronary heart disease (CHD) and premature mortality [2]. Besides resulting in macrovascular complications, there is growing evidence that MS, like diabetes mellitus, causes microvascular complications in patients with type 2 diabetes mellitus [3-5]. Nearly 70-80% of the population with diabetes mellitus is diagnosed with MS [6-8].

The correlation between MS and macro- and microvascular complications, in patients with diabetes mellitus, has been shown previously in American and European subjects [9-11]. The data in the Asian population is scanty and controversial. In Japan, the Funagata Study observed that MS, using the definition of the International Diabetes Federation (IDF), was associated with microvascular changes in the retina [12]. However, Terauchi *et al *[13] found that neither the presence of MS (as defined by the IDF guideline) nor an increased waist circumference increased the risk of micro- or macrovascular complications in Japanese patients with type 2 diabetes mellitus.

The Asian population is somewhat different from the Caucasian population. The incidence of CHD and the absolute risk of death from CHD, at the same level of blood pressure, is lower in Asians, more so in populations with diabetes [14,15]. The prevalence of obesity and its impact on cardiovascular disease is also different in Asians, compared to Caucasians [16].

The present population-based study aims to find out the prevalence of MS in the Indian population with type 2 diabetes mellitus in relation to gender, duration of diabetes, and to evaluate the influence of MS and its individual components on microvascular complications such as diabetic retinopathy (DR), diabetic nephropathy and diabetic neuropathy.

## Methods

The sample population was recruited from the SN-DREAMS 1 study which was a population-based cross-sectional study. The methodology has been described elsewhere [17]. The study population was selected by multistage systematic random sampling. The sampling was done in two stages: selection of divisions using computer generated random numbers, and selection of study subjects randomly from each selected division. A target sample size was calculated based on the following assumptions: the prevalence of diabetic retinopathy was assumed to be 1.3%, with a relative precision of 25%, a drop out rate of 20% and a design effect of 2. The sampling was stratified based on the socio-economic criteria. Ten divisions were selected and 600 individuals were enumerated from each division to meet the target. Thus, 5830 subjects from the general population aged ≥ 40 years were enumerated.

Of the 5830 subjects enumerated, 1414 subjects with diabetes (both known and newly diagnosed) were analyzed for the study (the response rate for the first fasting blood sugar estimation was 96.20%; the response rate for the base hospital examination was 85.60%; 8.7% were non-diabetic, after the second blood sugar estimation, and 0.78% retinal images were un-gradable). Subjects with diabetes were identified based on the American Diabetes Association criteria and they underwent a detailed examination at the base hospital [18].

After eight hours of overnight fasting, the fasting blood sample was taken to estimate the plasma glucose and serum lipids. For those with provisional diabetes, confirmation of diabetes was done by re-estimation of fasting blood glucose by enzymatic assay; glucose was oxidized by glucose oxidase to produce gluconate and hydrogen peroxide, which was then detected photometrically. A biochemical analysis was done on Merck Micro Lab 120, semi automated analyzer. The total serum cholesterol (CHOD-POD method), HDL (after protein precipitation CHOD-POD method) and serum triglycerides (CHOD-POD) were estimated.

Anthropometric measurements including weight, height, waist and hip measurements were obtained using standardized techniques. The blood pressure was recorded, in the sitting position, in the right arm to the nearest 2 mm Hg using the mercury sphygmomanometer (Diamond Deluxe BP apparatus, Pune, India). Two readings were taken, five minutes apart, and their mean was taken as the blood pressure.

The study was approved by the Institutional Review Board, and a written informed consent was obtained from the subjects as per the Helsinki declaration [19].

### Definitions

#### Metabolic syndrome

MS was defined using the International Diabetes Federation (IDF) consensus group guidelines as abdominal obesity (waist circumference ≥ 90 cm for men and ≥ 80 cm for women) plus two or more of the following risk factors: blood pressure ≥ 130/85 mm Hg, fasting plasma glucose ≥ 5.6 mmol/l (100 mg/dl) or pre-existing diabetes, serum triglycerides ≥ 1.7 mmol/l (≥ 150 mg/l) and HDL levels < 1.03 mmol/l (< 40 mg/dl) for men and < 1.29 mmol/l (< 50 mg/dl) for women [20].

#### Diabetic Retinopathy

All patients had their fundi photographed using the 45° four-field stereoscopic digital photography. The diagnosis of DR was based on the modified Klein classification (Modified Early Treatment Diabetic Retinopathy Study scales) [21]. DR was divided into mild, moderate and severe non-proliferative diabetic retinopathy (NPDR) and proliferative diabetic retinopathy (PDR); clinically significant macular edema (CSME) was graded as absent or present [22]. This grading was done by two independent observers in a masked fashion; the grading agreement was high (k = 0.83) [17].

#### Diabetic Nephropathy

The microalbuminuria was estimated, using the first morning urine sample, by a semi-quantitative procedure (Clintek 50 Bayer Urine Analyzer). Subjects were considered to have microalbuminuria, if the Albumin Creatinine Ratio (ACR) was between 30 and 299 mg/g [23].

#### Diabetic Neuropathy

Diabetic neuropathy assessment was done by measuring the Vibration Perception Threshold (VPT) using a sensitometer. The VPT was measured, by a single observer, by placing a biothesiometer probe perpendicular to the distal plantar surface of the great toe of both the legs. The VPT was measured at a voltage level when the patient felt the first vibration sensation. The mean VPT measure of the three readings, of both the legs, was considered for the analysis. The presence of diabetic neuropathy was considered if the VPT value was > 20 V [24].

### Statistical analysis

A computerized database was created for all the records. A statistical package for the Social Sciences-SPSS (version 9.0) was used for statistical analysis. All the data were expressed as mean ± S.D or as percentage. The statistical significance was assumed at *p *value ≤ 0.05. Univariate and multivariate logistic regression analyses were performed to elucidate the risk factors for microangiopathies. The Odds Ratio (OR), with 95% confidence intervals, was calculated for the studied variables.

## Results

The prevalence of MS in our subjects, with type 2 diabetes mellitus, was 73.8% (95% CI 71.5-76.0); the age and gender adjusted prevalence being 73.3% (95% CI 73.2-73.3). The prevalence was higher in women 83.3% (95% CI 80.4-86.1) compared to men 65.3% (95% CI 61.9-68.7). Table 1 shows the prevalence of MS in relation to gender and the duration of diabetes in subjects with type 2 diabetes mellitus. The prevalence of MS in men decreased with an increase in the duration of diabetes (67.4%, 59.7% and 39.1% in duration < 10 years, 10-20 years and > 20 years respectively, trend p = 0.002) whereas it remained almost similar in women (83.8%, 79.1% and 81.8%, trend p = 0.401).

**Table 1:** Prevalence of Metabolic Syndrome in relation to gender and duration of Diabetes in the study population

Duration of Diabetes	Prevalence of Metabolic Syndrome			
		
(Years)	n	%	(95% CI)	Trend *p*
**Overall**				
0-10 (N = 1194)	901	75.5	[73.0-77.9]	**0.0002**
11-20 (N = 186)	124	66.7	[59.9-73.4]	
> 20 (N = 34)	18	52.9	[36.2-69.7]	
Total (N = 1414)	1043	73.8	[71.5-76.0]	
**Men**				
0-10 (N = 608)	410	67.4	[63.7-71.2]	**0.002**
11-20 (N = 119)	71	59.7	[50.8-68.5]	
> 20 (N = 23)	9	39.1	[19.2-59.1]	
Total (N = 750)	490	65.3	[61.9-68.7]	
**Women**				
0-10 (N = 586)	491	83.8	[80.8-86.8]	0.401
11-20 (N = 67)	53	79.1	[69.4-88.8]	
> 20 (N = 11)	9	81.8	[59.0-104.6]	
Total (N = 664)	553	83.3	[80.4-86.1]	

N, Total population; n, Population with metabolic syndrome.

Table 2 shows the age and gender adjusted prevalence of individual components of MS in subjects with type 2 diabetes mellitus. The prevalence of increased waist circumference was 75.5% (95% CI 75.4-75.6); of hypertension, 75.3%, (95% CI 75.2-75.3%); of elevated serum triglycerides, 37.5% (95% CI 37.5-37.6); and of reduced serum HDL, 71.3% (95% CI 71.2-71.3%).

**Table 2:** Prevalence of individual components of Metabolic Syndrome

	Prevalence of Metabolic Syndrome			
Components	N	n	% *	[95% of CI]*
Increased Waist circumference	1414	1077	75.5	[75.4-75.6]
Hypertension	1414	1026	75.3	[75.2-75.3]
Elevated serum triglyceride	1414	547	37.5	[37.5-37.6]
Reduced serum HDL-Cholesterol	1414	1026	71.3	[71.2-71.3]

>N, Total population; n, Population with metabolic syndrome;

Age and gender adjusted

Table 3 shows the baseline demographic and laboratory characteristics of the study population. Overall, subjects with MS had a higher serum non HDL cholesterol (p = 0.029). However, they had lesser duration of diabetes than those without MS (p < 0.0001). On evaluating men and women separately, men with MS showed similar findings except for a raised level of hemoglobin in subjects with MS (p = 0.013). But in women with MS, the duration of diabetes and serum non HDL cholesterol were not significantly different (p = 0.084 and 0.488 respectively), and the levels of total serum cholesterol and LDL cholesterol were lower when compared to subjects without MS (p = 0.048 and 0.002 respectively). As observed in men, the hemoglobin level was significantly higher in women with MS (p = 0.003).

**Table 3:** Baseline characteristics of the study population

	Metabolic Syndrome								
	
Variables	Overall			Men			Women		
			
	Absent	Present	*p*	Absent	Present	*p*	Absent	Present	*p*
	n = 371	n = 1043		n = 260	n = 490		n = 111	n = 553	
Age (years)	57.0 ± 10.3	56.1 ± 9.9	0.119	57.1 ± 10.5	56.8 ± 10.5	0.664	56.8 ± 9.8	55.4 ± 9.4	0.181
HbA_1C _(%)	8.3 ± 2.4	8.2 ± 2.1	0.366	8.3 ± 2.4	8.2 ± 2.1	0.475	8.2 ± 2.6	8.1 ± 2.1	0.687
Duration of diabetes (years)	6.6 ± 7.5	5.1 ± 5.7	**< 0.0001**	7.1 ± 8.0	5.7 ± 6.0	**0.008**	5.5 ± 5.6	4.6 ± 5.3	0.084
Total serum cholesterol (mg/dl)	184.5 ± 40.9	187.2 ± 40.7	0.266	178.1 ± 38.4	183.2 ± 38.4	0.081	199.5 ± 42.8	190.8 ± 42.3	**0.048**
Serum LDL cholesterol (mg/dl)	113.2 ± 35.9	111.8 ± 35.2	0.506	106.8 ± 33.6	106.3 ± 33.8	0.845	128.2 ± 37.2	116.7 ± 35.8	**0.002**
Serum Non HDL cholesterol (mg/dl)	3.7 ± 1.0	3.8 ± 1.0	**0.029**	3.6 ± 0.9	3.8 ± 0.9	**0.009**	3.9 ± 1.0	3.9 ± 1.1	0.488
Hemoglobin (gm/dl)	13.8 ± 1.8	13.8 ± 1.4	0.884	14.2 ± 1.9	14.5 ± 1.4	**0.013**	12.7 ± 1.3	13.1 ± 1.1	**0.003**

HbA_1C _(%), Glycosylated hemoglobin; LDL, Low density lipoprotein; HDL, High-density lipoprotein

Table 4 evaluates the effect of MS on the prevalence of microvascular complications of diabetes. In subjects with diabetes mellitus, without and with MS, the prevalence of retinopathy was 21.3% and 16.9% (p = 0.057); prevalence of nephropathy was 20.5% and 18.0% (p = 0.296) and prevalence of neuropathy was17.2% and 19.4% (p = 0.353) respectively. When evaluated separately, in men and women, the diagnosis of MS was not significantly associated with the presence of microvascular complications in our study population except for a lower prevalence of nephropathy in men with MS (p = 0.029).

**Table 4:** Comparison of prevalence of microangiopathies in the study population with and without Metabolic Syndrome

Metabolic Syndrome	Diabetic Retinopathy, n = 255	*p*	Diabetic Nephropathy, n = 264	*p*	Diabetic Neuropathy, n = 264	*p*
**Overall**						
Absent (n = 371)	79 (21.3)	0.057	76 (20.5)	0.296	63 (17.2)	0.353
Present (n = 1043)	176 (16.9)		188 (18.0)		201 (19.4)	
**Men**						
Absent (n = 260)	61 (23.5)	0.241	60 (23.1)	**0.029**	46 (17.9)	0.304
Present (n = 490)	97 (19.8)		81 (16.5)		103 (21.1)	
**Women**						
Absent (n = 111)	18 (16.2)	0.599	16 (14.4)	0.222	17 (15.6)	0.556
Present (n = 553)	79 (14.3)		107 (19.3)		98 (17.9)	

Table 5 shows the effect of aggregation of more number of components of MS on the prevalence of microangiopathies. The IDF definition of MS requires central obesity to be the essential criterion. Since our whole study population was diabetic, the presence of diabetes or raised plasma glucose also became an unchangeable criterion in our study cohort. To study the desired outcome, we grouped the patients into three sets, in increasing order of presence of three, four or five criteria of MS. When patients were grouped in such a way, the prevalence of diabetic retinopathy, overall and in men, was not different. In women, however, the prevalence of retinopathy increased with a rise in the number of MS components (12.6%, 20.1% and 16.4%, trend p = 0.193 overall; 17.5%, 22.8% and 21.6%, trend p = 0.779 in men; 6.5%, 18.0% and 15.0%, trend p = 0.049 in women). Overall and in women, the clustering of MS components led to an increase in the prevalence of diabetic nephropathy. However, there was no such trend in men (13.9%, 18.5% and 21.7%, trend p = 0.013 overall; 11.6%, 19.2% and 26.3%, trend p = 0.001 for women; 15.8%, 17.6% and 15.9%, trend p = 0.945 in men). For diabetic neuropathy, the prevalence was not different overall and in men, but it increased with grouping of MS components in women (21.2%, 16.8% and 21.6%, trend p = 0.929 overall; 26.9%, 17.1% and 19.2%, trend p = 0.077 in men and 14.0%, 16.6% and 23.6%, trend p = 0.003 in women).

**Table 5:** Effect of clustering of components of Metabolic Syndrome on microangiopathies

		Prevalence of Microangiopathies								
		
Components of MS		Diabetic Retinopathy			Diabetic Nephropathy			Diabetic Neuropathy		
		
	N	n	%	Trend *p*	n	%	Trend *p*	n	%	Trend *p*
**Overall **(n = 1043)										
+ Any one	309	39	12.6	0.193	43	13.9	**0.013**	65	21.2	0.929
+Any two	448	90	20.1		83	18.5		75	16.8	
+ Any three	286	47	16.4		62	21.7		61	21.6	
**Men **(n = 490)										
+ Any one	171	30	17.5	0.779	27	15.8	0.945	46	26.9	0.077
+Any two	193	44	22.8		34	17.6		33	17.1	
+ Any three	126	23	18.3		20	15.9		24	19.2	
**Women **(n = 553)										
+ Any one	138	9	6.5	**0.049**	16	11.6	**0.001**	19	14.0	**0.030**
+Any two	255	46	18.0		49	19.2		42	16.6	
+ Any three	160	24	15.0		42	26.3		37	23.6	

MS, Metabolic Syndrome; MS absent, Diabetes only or Diabetes + Obesity; + Any one, Diabetes + Obesity + Any of hypertension, reduced HDL or elevated triglyceride; + Any two, Diabetes + Obesity + Any two of hypertension, reduced HDL or elevated triglyceride; + Any three, Diabetes + Obesity + All three of hypertension, reduced HDL or elevated triglyceride; N, Total population with metabolic syndrome; n, Population with metabolic syndrome having microangiopathies

Table 6 shows the influence the duration of diabetes has on the prevalence of microangiopathies in patients with and without MS. The prevalence of retinopathy and neuropathy in MS subjects, who had diabetes for < 10 years, was more in both men and women; it was more in women but not in men when the duration of diabetes varied from 11-20 years; and when the duration of diabetes was > 20 years, it was not different in men or women when compared to subjects without MS. Nephropathy was more prevalent in women with MS and whose duration of diabetes was < 10 years and 11-20 years but the prevalence was not different when the duration of diabetes was > 20 years.

**Table 6:** Effect of duration of Diabetes on the prevalence of microangiopathies in study population with or without Metabolic Syndrome

		Prevalence of Microangiopathy					
		
Variables	Groups	Duration (0-10 years)	*p*	Duration (11-20 years)	*p*	Duration (> 20 years)	*p*
**Overall**							
Retinopathy	MS Absent	48 (28.2)	**< 0.0001**	25 (34.7)	**0.013**	6 (46.2)	1.000
	MS Present	122 (71.8)		47 (65.3)		7 (53.8)	
							
Nephropathy	MS Absent	61 (29.6)	**< 0.0001**	12 (22.6)	**< 0.0001**	3 (60.0)	1.000
	MS Present	145 (70.4)		41 (77.4)		2 (40.0)	
							
Neuropathy	MS Absent	38 (19.9)	**< 0.0001**	18 (31.0)	**0.005**	7 (46.7)	1.000
	MS Present	153 (80.1)		40 (69.0)		8 (53.3)	
**Men**							
Retinopathy	MS Absent	36 (34.3)	**0.002**	19 (43.2)	0.451	6 (66.7)	0.508
	MS Present	69 (65.7)		25 (56.8)		3 (33.3)	
							
Nephropathy	MS Absent	46 (43.4)	0.206	11 (36.7)	0.200	3 (60.0)	1.000
	MS Present	60 (56.6)		19 (63.3)		2 (40.0)	
							
Neuropathy	MS Absent	24 (24.2)	**< 0.0001**	16 (40.0)	0.268	6 (60.0)	0.754
	MS Present	75 (75.8)		24 (60.0)		4 (40.0)	
**Women**							
Retinopathy	MS Absent	12 (18.5)	**< 0.0001**	6 (21.4)	**0.004**	0 (0.0)	0.125
	MS Present	53 (81.5)		22 (78.6)		4 (100.0)	
							
Nephropathy	MS Absent	15 (15.0)	**< 0.0001**	1 (4.3)	**< 0.0001**	0 (0.0)	NA
	MS Present	85 (85.0)		22 (95.7)		0 (0.0)	
							
Neuropathy	MS Absent	14 (15.2)	**< 0.0001**	2 (11.1)	**0.001**	1 (20.0)	0.375
	MS Present	78 (84.8)		16 (88.9)		4 (80.0)	

MS, Metabolic Syndrome

To evaluate the relation between the presence of diabetic microvascular complications and individual MS components, we performed a multiple logistic regression analysis by adjusting variables such as age, gender, smoking and alcohol (Table 7). The results showed that the independent risk factors for retinopathy were glycosylated hemoglobin - OR 1.16 (95% CI 1.07-1.26) and duration of diabetes - OR 1.13 (95% CI 1.09-1.16). For nephropathy, the risk factors were glycosylated hemoglobin - OR 1.24 (95% CI 1.15-1.34), duration of diabetes - OR 1.04 (95% CI 1.01-1.07) and hypertension - OR 1.80 (95% CI 1.20-2.70). For neuropathy, the independent risk factors came out to be reduced serum HDL cholesterol - OR 1.03 (95% CI 1.01-1.05) and increased waist circumference - OR 1.02 (1.00-1.04).

**Table 7:** Multiple logistic model for risk factors for microangiopathies

	Overall		Men		Women	
	
*Independent variables*	OR (95% CI)	*p*	OR (95% CI) *	*p*	OR (95% CI) ^†^	*p*
**Retinopathy**						
Glycosylated Hemoglobin	1.16 (1.07-1.26)	**< 0.0001**	1.14 (1.02-1.28)	**0.020**	1.19 (1.07-1.34)	**0.002**
Duration of Diabetes	1.13 (1.09-1.16)	**< 0.0001**	1.13 (1.08-1.17)	**< 0.0001**	1.13 (1.08-1.18)	**< 0.0001**
Hypertension	1.18 (0.80-1.77)	0.392	1.09 (0.65-1.82)	0.746	1.41 (0.74-2.69)	0.297
Elevated serum Triglyceride	1.01 (0.99-1.00)	0.560	1.00 (0.99-1.00)	0.256	1.00 (0.99-1.00)	0.857
Reduced serum HDL-Cholesterol	1.01 (0.99-1.03)	0.254	1.03 (1.00-1.06)	**0.038**	0.99 (0.97-1.02)	0.618
Increased Waist circumference	0.98 (0.96-1.01)	0.188	0.96 (0.93-0.99)	**0.037**	1.01 (0.98-1.04)	0.663
**Nephropathy**						
Glycosylated Hemoglobin	1.24 (1.15-1.34)	**< 0.0001**	1.19 (1.07-1.34)	**0.002**	1.27 (1.15-1.39)	**< 0.0001**
Duration of Diabetes	1.04 (1.01-1.07)	**0.008**	1.04 (1.00-1.08)	0.052	1.04 (0.99-1.08)	0.076
Hypertension	1.80 (1.20-2.70)	**0.004**	1.50 (0.86-2.63)	0.155	2.17 (1.19-3.93)	**0.011**
Elevated serum Triglyceride	1.00 (0.99-1.00)	0.976	0.99 (0.99-1.00)	0.661	1.00 (0.99-1.00)	0.473
Reduced serum HDL-Cholesterol	0.99 (0.98-1.02)	0.857	1.01 (0.98-1.04)	0.612	0.99 (0.97-1.01)	0.450
Increased Waist circumference	1.02 (1.00-1.04)	0.055	1.03 (0.99-1.07)	0.064	1.01 (0.99-1.04)	0.289
**Neuropathy**						
Glycosylated Hemoglobin	1.05 (0.97-1.14)	0.192	1.08 (0.96-1.21)	0.198	1.03 (0.92-1.15)	0.596
Duration of Diabetes	1.02 (0.99-1.05)	0.181	1.01 (0.97-1.05)	0.653	1.03 (0.98-1.07)	0.215
Hypertension	1.19 (0.80-1.76)	0.384	0.87 (0.51-1.48)	0.607	1.83 (0.98-3.44)	0.059
Elevated serum Triglyceride	1.00 (0.99-1.00)	0.381	1.00 (0.99-1.00)	0.313	1.00 (0.99-1.00)	0.496
Reduced serum HDL-Cholesterol	1.03 (1.01-1.05)	**0.001**	1.06 (1.03-1.09)	**< 0.0001**	1.00 (0.98-1.03)	0.806
Increased Waist circumference	1.02 (1.00-1.04)	**0.043**	1.01 (0.97-1.04)	0.640	1.03 (1.00-1.06)	0.027

* All factors adjusted with age, gender, glycosylated hemoglobin, hemoglobin, socioeconomic status, smoking status and alcohol status

^† ^All factors adjusted with age, glycosylated hemoglobin, hemoglobin, socioeconomic status, smoking status and alcohol status

## Discussion

The present study reports an alarmingly high prevalence of MS, using IDF criteria, in subjects with type 2 diabetes mellitus in a population-based study. One of the IDF criteria for MS, the increased fasting plasma glucose ≥ 100 mg/dl or pre-existing diabetes, was present in all of the participants of the study; therefore, the "metabolic syndrome" in this study is not exactly the same as MS in general sense. In our cohort, almost every three out of four subjects with type 2 diabetes had MS. A similar high prevalence was reported by Surana *et al *[25] in urban Indian population with type 2 diabetes (77.2%) and by Foucan *et al *[26] in Indian immigrants in the USA with diabetes (77%). This prevalence of MS in the South Indian population with diabetes is more than two-fold higher than the reported prevalence in the general urban Indian population [27]. Using a similar definition as ours (IDF), Terauchi *et al *[13] and Bonadonna *et al *[11] reported the prevalence of MS to be 58.5% and 77.6% respectively, which was comparable to that observed in our study.

The present study has observed gender-wise differences with respect to prevalence of MS and its influence on microangiopathies. The MS, in the defined population of type II diabetes mellitus, was more prevalent in women than men (Table 1); there was a positive correlation between the number of components of MS and all types of microangiopathies (Table 5) in women. Like our study, Ghani *et al *[28] and Bonadonna *et al *[11] also reported a higher prevalence of MS in women; however Shimajiri *et al *[4] reported a higher prevalence in men. Among the Indian diabetic population, a higher prevalence of MS in women was reported by Surana *et al *[25]. They proposed the cause to be a higher prevalence of low HDL and central obesity in women, which could partially be attributed to the lower cut-off for waist circumference and higher cut-off for HDL in women as compared to men. In our study, we also noted a higher BMI and lower HDL cholesterol in women as compared to men. But the waist circumference was lower in women despite the lower cut-off, which suggests that there are other factors which increase the prevalence of MS in women. Bonadonna *et al *[11] also commented in their study that this trend could be both due to a true biological phenomenon or a suboptimal choice of gender-specific diagnostic thresholds. Because of these gender-wise differences, female gender should become a focus of high risk target-screening, and attempts should be made to normalize each component of MS so as to minimize the risk of microangiopathies.

Shimajiri *et al *[4] reported that the prevalence of MS decreases along with an increase in the duration of diabetes. They suggested the possible reason to be a decreased BMI as a result of medical intervention in the lifestyle of long term diabetic patients. In our study, we noted this trend only in men. We feel that the better accessibility to medical care and more health awareness among men in our country can be a factor responsible for this. Whether there are other biological or hormonal factors playing a role is not known. We also observed different trends in obesity indices in men and women in the same study cohort, which is possibly related to postmenopausal hormonal changes in women [29]. This might also explain the gender differences in the current study.

Similar to our finding, Ghani *et al *[28] also found an association of MS with less duration of diabetes. The occurrence of microangiopathies in diabetes mellitus is influenced by several of the components of the MS; this implies a possible relationship between microangiopathies and MS. The association of MS with microvascular complications of type 2 diabetes i.e. diabetic retinopathy, nephropathy and neuropathy has been studied in many reports [4,9,11,28], They found the increased prevalence of all three complications in patients with diabetes using different definitions of MS. Kawasaki *et al *[12] used the IDF criteria and reported that the associations between MS and retinal microvascular signs appeared mainly driven by the associations with larger waist circumference. Isomaa *et al *[5] reported that MS was strongly associated with microalbuminuria but had little effect on the development of retinopathy and neuropathy. However, a study in Japanese patients, on the other hand, did not show any statistically significant association of MS with the prevalence of microvascular complications in type 2 diabetes mellitus [13]. They attributed it to possible ethnicity related differences because in Asian population, MS has also been reported to be non predictive of coronary artery disease in contrast to the results in Caucasian population. In our study, we found a similar lack of association between MS and the increased prevalence of microvascular disease.

Some previous studies have reported an increase in the prevalence of microangiopathies when patients were grouped according to the number of MS components [9,28,30]. In men, we found that there was no trend of increase in diabetic microvascular complications with an increase in the MS features. However, in women, there was a statistically significant increase in the prevalence of diabetic microangiopathies with an increase in the MS components.

On evaluating the influence of the duration of diabetes on the prevalence of microangiopathies in patients with MS, we found that there was a significant association between MS and the prevalence of microangiopathies in subjects with type 2 diabetes mellitus in the early course of the disease which disappeared with the increasing duration of diabetes in both men and women. This suggests that MS might have a more significant influence on increasing the prevalence of microvascular disease in recent diabetics, but gradually with increasing duration of diabetes, this influence decreases. One possible explanation for this could be better metabolic control in diabetic patients due to increasing awareness with longer duration of diabetes. We did find this trend in the present study; subjects with longer duration of diabetes (> 20 years) had better metabolic control (FBS< 110 mg %) than those with shorter duration of diabetes (0-10 and 11-20 years). The FBS < 110 mg% was noted in 61.8%, 73.7% and 79.2% of subjects with duration of diabetes mellitus being 0-10 years, 11-20 years and > 20 years, respectively (*p *= 0.005).

We found the risk factors for retinopathy to be glycosylated hemoglobin and duration of diabetes, for nephropathy to be glycosylated hemoglobin, duration of diabetes, hypertension, increased body mass index and increased waist circumference and for neuropathy to be reduced serum HDL cholesterol on multivariate analysis. Different associations have also been reported in previous studies [5,12,13]. We also found an association of waist circumference with nephropathy as was reported by Hanai *et al *[30].

## Conclusions

The strength of current study is that it was a population based study that used standardized methods for evaluation of microvascular complications of diabetes mellitus. However, the causal relationship could not be assessed because of the cross sectional nature of study. The prevalence and risk factors of microangiopathies among subjects with MS may also be influenced by presence of diabetes mellitus.

This study reports the prevalence and risk factors for MS in subjects with type 2 diabetes. The gender differences in the prevalence and the risk factors for MS in the population with diabetes need further confirmation in a larger prospective study.

## Competing interests

The authors declare that they have no competing interests.

## Authors' contributions

RR participated in acquisition of data and drafting the manuscript. AG and KV participated in writing the manuscript. SSP and SG participated in data collection and analysis. VK performed the statistical analysis. TS participated in study design and gave final approval of the version to be published. All authors have read and approved the final manuscript.
